# Medical identity; perspectives of students from two countries

**DOI:** 10.1186/s12909-020-02351-7

**Published:** 2020-11-10

**Authors:** Conor Gilligan, Teresa Loda, Florian Junne, Stephan Zipfel, Brian Kelly, Graeme Horton, Anne Herrmann-Werner

**Affiliations:** 1 School of Medicine and Public Health, University of Newcastle, Callaghan, Australia; 2 Medical Department VI/Psychosomatic Medicine and Psychotherapy, University Hospital Tuebingen, Osianderstr. 5, D-72076 Tuebingen, Germany; 3 School of Medicine, University of Tuebingen, Tuebingen, Germany

**Keywords:** Identity, Doctor, Medical students, Medical curriculum

## Abstract

**Background:**

The development of professional identity is a fundamental element of medical education. There is evidence that in Germany, students’ perceptions of the ideal and real doctor differ, and that of themselves as physicians falls between these constructs. We sought to compare students’ perceptions of themselves, the ideal doctor, and the ‘real’ doctor and investigate differences from first to final year in the relationships between these constructs, as well as differences between Australian and German cohorts.

**Method:**

Students in the first and final years of their medical program at one Australian and one German university were invited to complete the Osgood and Hofstatter polarity profile, involving the description of their mental image of the ideal and real doctor, and the doctor they hope to become, with adjectives provided.

**Results:**

One hundred sixty-seven students completed the survey in Australia (121 year 1, 46 year 5) and 188 in Germany (164 year 1, 24 year 6). The perception of the ideal doctor was consistent across all respondents, but that of the real doctor and self-image differed between country and year. Differences existed between country cohorts in perceptions of ‘confidence’, ‘strength’, ‘capability’ and ‘security’.

**Conclusions:**

The pattern previously reported among German students was maintained, but a different pattern emerged among Australian students. Differences between countries could reflect cultural differences or variations in the overt and hidden curricula of medical schools. Some of the constructs within the profiles are amenable to educational interventions to improve students’ confidence and sense of capability.

## Background

For medical schools in many parts of the world, admissions processes aim to select students who are not only academically capable, but who possess important skills and values such as altruism, teamwork, and communication [1, 2]. While these attributes are regarded as important for entry into medical school, and thus the profession, there is evidence that students’ idealism [3, 4], ethical self-identity [5–7] and their empathy and patient centred communication skills [8] decline over time. This leads to a questioning of how medical students perceive these attributes among their profession and how medical schools help to mould students’ professional identities.

Medical students often enter the medical profession with aspirations about providing care and ‘helping people’. Influences upon students throughout medical school include their own ideals and stereotypes, their academic teachers and clinical supervisors, and encounters with other physicians and patients. These influences can vary greatly, as evidenced by the vast literature relating to the hidden curriculum; the difference between students’ own values and what they are taught in their ‘on-campus’ learning, and the explicit or implicit messages they are exposed to in clinical environments [9].

In recent years, medical program accrediting bodies internationally have required medical undergraduate and postgraduate curricula to include teaching and assessment of professionalism. Cruess et al. describe the objective of teaching medical professionalism as being to assist learners to develop professional identities [10]. Two important constructs intersect here; that of professionalism and what constitutes professional and ethical behaviour, and that of identity, as an individual and a professional. Identity can be considered at the level of the individual (personal core values and culture), the role (as a medical student, an intern etc.), and the profession (a group member as a part of the medical profession) [11]. Each of these levels inter-relate and change over time. In the case of medical professional identity, the physician that students ultimately ‘become’ is the result of a gradual process of development [12].

The nature of medical education, including the hidden curriculum, varies in different medical schools and different countries, with the hidden curriculum being a culturally influenced phenomenon [7, 13]. Indeed, the hidden curriculum has been defined as the *‘cultural mores that are transmitted, but not openly acknowledged, through formal and informal educational endeavours’*. [14] Hafler et al. extended the hidden curriculum beyond the concept of students as recipients of a faculty-led curriculum to propose that faculty are also exposed to a hidden curriculum through approaches to faculty development and the organisational context into which they are enculturated [9, 13]. Hidden curricula messages to students are likely to be negatively influenced when teaching faculty feel that the teaching element of their work is not highly valued. Thus medical education and the development of medical professional identities is a complex process of socialisation likely to change over time and differ across schools and jurisdictions.

A semantic differential approach has been used to address the challenge of measuring emotions, motivation and attitude [15]. This approach involves a process in which associative meanings of words are represented by rating of bipolar pairs of adjectives [16]. Hofstätter and Lübbert coined the term “polarity profile” in their attempt to depict comparable stereotypes as a further development of Osgood’s semantic differential [17]. Applied to the medical setting, Speierer was the first to measure German medical undergraduate students’ images of the real and ideal doctor as well as their self-image using 18 pairs of adjectives derived from a large patient survey [18]. Around 20 years later, Schrauth et al. repeated the survey using the same pairs of adjectives [19]. The results of these two studies indicate that the students’ perceptions of the ideal doctor have remained relatively constant over time, but that there is a substantial deviation between this perception and that of the ‘real’ doctor as well as their self-perception [19]. In general, the ideal doctor is characterised by a set of expectations which differ from the ‘real’ doctors encountered. This is consistent with the concept of the medical school culture and hidden curriculum shaping students’ perceptions of physicians as they advance through their training. To some extent, perceptions of the ‘ideal’ doctor are likely to be somewhat universal, but the realities of practice are more likely to differ with complex cultural nuances.

A pattern of differentiation between students’ perceptions of themselves, the ideal doctor and the real doctor has been established with students in Germany [18, 19]. This study sought to investigate whether the same pattern exists among students at an Australian medical school, and to explore the similarities and differences in the perceptions of students in first and final year cohorts in both countries. Specifically, we compare students’ perceptions of themselves, the ideal doctor, and the ‘real’ doctor and investigate differences from first to final year in the relationships between these constructs, as well as differences between Australian and German cohorts. The findings of this study will inform future work to understand cultural differences in medical education and the impacts of the medical education environment on students’ identity formation. Cross-cultural comparison provides insight into potential hidden curricula influences, offering a depth of understanding that cannot be gained through studying a single cultural context.

## Methods

In Australia, in February 2017, the incoming cohort of BMedSc/MD students (*n* = 131) at a regional university were invited to complete the polarity scale questionnaire (which was forward and back translated by Hahn, Herrmann-Werner and Junne 2014; personal communication) anonymously online as part of an orientation exercise on the first day of their program. Late in 2017, an invitation was sent via email to 197 students in their final year, who were asked to complete the instrument online during their last few weeks of medical school. Also, iPads were provided in registration and waiting rooms on the day of final Objective Structured Clinical Examinations to encourage completion. Completion of the instrument was regarded as implied consent.

In April 2018 the incoming cohort of medical students (*n* = 173) at a large German Medical Faculty as well as final year students (*n* = 42) attached to the University Hospital were approached within introductory lectures and invited to complete the polarity profile (paper-pencil version). All participants gave written consent and data collection was anonymous.

Herein, the two samples will be referred to as the Australian and German samples.

### Survey

The Osgood Hofstatter polarity profile was used in both English (Australia) and German (Germany). Respondents are asked to imagine an ‘ideal doctor’, consider the statement ‘in my opinion an ideal doctor is...’ and describe their mental image with the adjectives provided on a seven-point Likert scale. This question is repeated for ‘the real doctor’ and ‘the doctor you hope/plan to become’ (Australia) or ‘the way I really am’ (Germany). Differences in the self-image wording between cohorts was the result of a dual purpose of the survey in Australia; to explore professionalism and self-reflection. The survey takes less than 10 min to complete. The items (adjectives) and question are presented in the corresponding figures.

### Analysis

Descriptive statistics including mean values and associated standard deviations, frequencies, and percentages were used to summarise the demographic characteristics of participants. For the polarity profile, mean values and standard deviations were calculated for the sum score of all items and for the single items for ideal, real and self-image, and for each sub-group.

ANOVAS were used to test differences in demographic characteristics, and in ideal, real and self-image between the four groups. The level of significance was *p* < .05. Statistical analysis was performed using SPSS 25 (SPSS Incorporated, Chicago, IL). Data were normally distributed, as tested using the Kolmogorow-Smirnow-Test.

## Results

A total of 167 students completed the survey in Australia (121 in year 1 [92.4%] and 46 in year 5 [23.4%]) and 188 in Germany (164 in year 1 [94.8%] and 24 in year 6 [57.1%]). The average age of first year students was 21.3 (Standard Deviation [SD] 4.92) in Australia and 21.5 (SD 5.01) in Germany, and of final year students was 24.8 (SD 3.88) and 25.9 (SD 4.17) respectively. No significant age difference was found between the cohorts. In Australia, females made up just under half of the sample, while in Germany approximately 65% of the sample was female. This difference was significant for the first year cohort only (χ2 (1) = 7.020, *p* = .008). Some differences existed in the proportions of students who had undertaken previous training across all four cohorts (year 1 χ2(1) = 8.015, *p* = 0.005; year 5 χ2(1) = 3.892, *p* = 0.049). For detailed characteristics of the cohorts please see Table 1.

**Table 1:** Summary of participant demographic characteristics

	Australia Yr 1 (***n*** = 121)	Australia Yr 5 (***n*** = 46)	Germany Yr 1 (***n*** = 164)	Germany Year 6 (***n*** = 24)
Age Mean (SD)	21.29 (4.92)	24.78 (3.88)	21.52 (5.01)	25.92 (4.17)
Gender	Female: 60 (49.6%) Male: 59 (48.8%) Not specified: 2 (1.7%)	Female: 22 (47.8%) Male: 24 (52.2%)	Female: 106 (64.6%) Male: 58 (35.4%)	Female: 16 (66.7%) Male: 8 (33.3%)
Place of birth	Australia: 82 (67.8%) Other: 39 (32.2%) [Singapore: 18 (14.9%), India: 5 (4.1%), others very small numbers]	Australia: 32 (67.8%) Other: 14 (30.4%) [Singapore: 2 (4.3%), England: 3 (6.5%), others maximum *n* = 1]	Germany: 146 (89%) Other: 18 (11%) [Belgium: 2 (1.2%), Italy: 2 (1.2%), others maximum *n* = 1]	Germany: 21 (87.5%) Other: 3 (12.5%) [Belgium, Romania]
Other language at home	English: 107 (88.4%) English and other: 13 (10.7%)	English 100% English and other: 6 (13%)	German: 132 (80.5%) German and other: 24 (14.6%) Other: 8 (4.9%)	German: 21 (87.5%) German and other: 3 (12.5%) [English, Dutch, Polish]
Previous training	64 (52.9%)	9 (19.6%)	60 (36.6%)	11 (45.8%)
Parents physicians	28 (23.1%)	11 (23.9%)	29 (17.7%)	4 (16.7%)

**Fig. 1:**
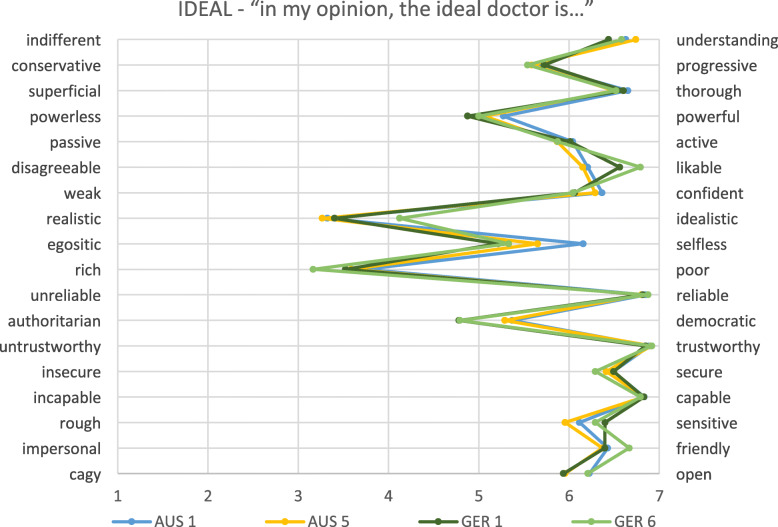
Physicians’ ideal image of all students separated to year level. *AUS = Australia; GER = Germany; numbers relate to year level*

**Fig. 2:**
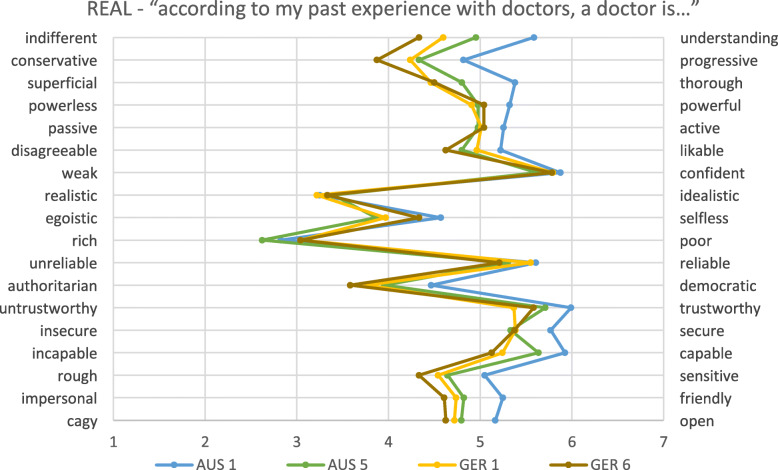
Physicians’ real image of all students separated to year level. *AUS = Australia; GER = Germany; numbers relate to year level*

**Fig. 3:**
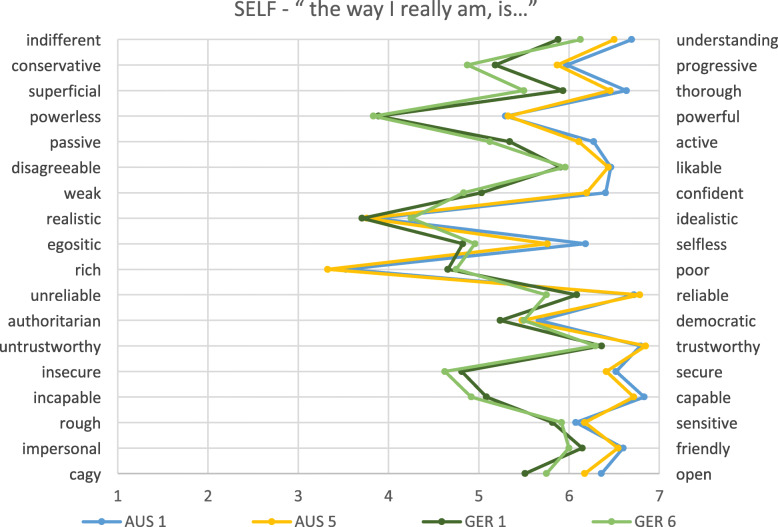
Physicians’ self image of all students separated to year level. *AUS = Australia; GER = Germany; numbers relate to year level*

In general, patterns were similar in the German and Australian cohorts, but the levels of ‘positivity’ or ‘negativity’ varied between the cohorts, as did the relationships between the constructs (see Figs. 1, 2, 3 and Tables 2, 3, 4).

**Table 2:** Summary of ratings of the ‘ideal’ physician

Likert scale (IDEAL)		Mean (SD)						
1	7	AUS yr1	AUS yr 5	Diff (p)	GER yr1	GER yr 5	Diff (p)	AUS vs GER difference (p)
Indifferent	Understanding	6.63 (0.59)	6.74 (0.57)	*p* > 0.05	6.44 (0.74)	6.58 (0.58)	*p* > 0.05	***p*** **< 0.01 F(1, 3.58) = 8.094**
Conservative	Progressive	5.64 (1.09)	5.58 (1.11)	*p* > 0.05	5.73 (0.99)	5.54 (1.02)	*p* > 0.05	*p* > 0.05
Superficial	Thorough	6.65 (0.72)	6.5 (0.62)	*p* > 0.05	6.60 (0.77)	6.52 (1.08)	*p* > 0.05	*p* > 0.05
Powerless	Powerful	5.27 (0.91)	5.07 (0.95)	*p* > 0.05	4.88 (0.99)	5.00 (0.98)	*p* > 0.05	***p*** **< 0.01 F(1, 9.186) = 10.01**
Passive	Active	6.04 (0.90)	5.87 (0.96)	*p* > 0.05	6.01 (0.98)	5.88 (0.85)	*p* > 0.05	*p* > 0.05
Disagreeable	Likeable	6.21 (0.90)	6.15 (0.92)	*p* > 0.05	6.56 (0.68)	6.79 (0.41)	*p* > 0.05	***p*** **< 0.01 F(1, 8.601) = 11.913**
Weak	Confident	6.37 (0.72)	6.29 (0.84)	*p* > 0.05	6.06 (0.94)	6.04 (0.77)	*p* > 0.05	***p*** **< 0.01 F(1,7.202) = 10.096**
Realistic	Idealistic	3.32 (1.39)	3.27 (1.27)	*p* > 0.05	3.40 (1.55)	4.13 (1.33)	***p*** **< 0.05 F(1, 10.931) = 4.706**	*p* > 0.05
Egoistic	Selfless	6.16 (0.88)	5.65 (1.10)	***p*** **< 0.01 F(1,8.5) = 9.495**	5.21 (1.09)	5.33 (1.09)	*p* > 0.05	***p*** **< 0.001 F(1, 55.089)**
Rich	Poor	3.74 (0.86)	3.59 (0.91)	*p* > 0.05	3.52 (0.98)	3.17 (0.92)	*p* > 0.05	***p*** **< 0.05 F(1, 4.122) = 4.750**
Unreliable	Reliable	6.85 (0.38)	6.80 (0.40)	*p* > 0.05	6.82 (0.49)	6.88 (0.34)	*p* > 0.05	*p* > 0.05
Authoritarian	Democratic	5.37 (1.19)	5.29 (1.16)	*p* > 0.05	4.78 (1.46)	4.79 (1.14)	*p* > 0.05	***p*** **< 0.001 F(1, 28.39)**
Untrustworthy	Trustworthy	6.89 (0.31)	6.91 (0.28)	*p* > 0.05	6.85 (0.42)	6.92 (0.28)	*p* > 0.05	*p* > 0.05
Insecure	Secure	6.49 (0.65)	6.41 (0.83)	*p* > 0.05	6.49 (0.75)	6.29 (0.91)	*p* > 0.05	*p* > 0.05
Incapable	Capable	6.83 (0.40)	6.86 (0.44)	*p* > 0.05	6.83 (0.41)	6.79 (0.41)	*p* > 0.05	*p* > 0.05
Rough	Sensitive	6.12 (0.84)	5.96 (1.03)	*p* > 0.05	6.40 (0.82)	6.29 (0.69)	*p* > 0.05	***p*** **< 0.01 F(1, 8.601) = 11.913**
Impersonal	Friendly	6.43 (0.75)	6.37 (0.80)	*p* > 0.05	6.40 (0.91)	6.67 (0.56)	*p* > 0.05	*p* > 0.05
Cagy	Open	6.23 (0.83)	5.96 (0.95)	*p* > 0.05	5.94 (1.17)	6.21 (0.72)	*p* > 0.05	*p* > 0.05

*AUS* Australia, *GER* Germany

**Table 3:** Summary of ratings of the ‘real’ physician

Likert scale (REAL)		Mean (SD)						
1	7	AUS yr1	AUS yr 5	Diff (p)	GER yr1	GER yr 5	Diff (p)	AUS vs GER difference (p)
Indifferent	Understanding	5.59 (0.91)	4.96 (1.13)	***p*** **< 0.001 F(1, 13.069) = 13.806**	4.60 (1.30)	4.33 (1.01)	*p* > 0.05	***p*** **< 0.001 F(1, 63.969) = 48.26**
Conservative	Progressive	4.82 (1.13)	4.33 (1.09)	***p*** **< 0.05 F(1, 7.711) = 6.199**	4.24 (1.13)	3.88 (0.85)	*p* > 0.05	***p*** **< 0.001 F(1, 21.623) = 17.345**
Superficial	Thorough	5.38 (1.08)	4.80 (0.99)	***p*** **< 0.01 F(1, 11.041) = 9.856**	4.46 (1.36)	4.50 (1.14)	*p* > 0.05	***p*** **< 0.001 F(1, 50.227) = 33.640**
Powerless	Powerful	5.32 (0.95)	4.98 (1.29)	*p* > 0.05	4.91 (1.17)	5.04 (0.81)	*p* > 0.05	***p*** **< 0.05 F(1, 8.115) = 6.714**
Passive	Active	5.26 (1.08)	4.98 (1.01)	*p* > 0.05	5.01 (1.19)	5.04 (0.86)	*p* > 0.05	*p* > 0.05
Disagreeable	Likeable	5.22 (0.93)	4.80 (0.87)	***p*** **< 0.01 F(1, 5.873) = 7.073**	4.96 (1.21)	4.63 (1.13)	*p* > 0.05	*p* > 0.05
Weak	Confident	5.88 (0.72)	5.62 (0.96)	*p* > 0.05	5.79 (0.95)	5.78 (0.67)	*p* > 0.05	*p* > 0.05
Realistic	Idealistic	3.25 (1.46)	3.40 (1.19)	*p* > 0.05	3.22 (1.31)	3.33 (1.20)	*p* > 0.05	*p* > 0.05
Egoistic	Selfless	4.57 (1.26)	3.87 (1.20)	***p*** **< 0.01 F(1, 16.237) = 10.449**	3.97 (1.15)	4.33 (0.96)	*p* > 0.05	***p*** **< 0.01 F(1, 11.652) = 8.042**
Rich	Poor	2.83 (1.01)	2.62 (1.11)	*p* > 0.05	3.04 (1.14)	3.04 (0.86)	*p* > 0.05	***p*** **< 0.05 (1, 6.497) = 5.647**
Unreliable	Reliable	5.61 (0.94)	5.30 (0.91)	*p* > 0.05	5.55 (4.14)	5.21 (1.06)	*p* > 0.05	*p* > 0.05
Authoritarian	Democratic	4.47 (1.32)	3.96 (1.04)	***p*** **< 0.05 F(1, 8.549) = 5.448**	3.77 (1.33)	3.58 (1.10)	*p* > 0.05	***p*** **< 0.001 F(1, 29.957) = 18.153**
Untrustworthy	Trustworthy	5.99 (0.82)	5.71 (0.79)	*p* > 0.05	5.37 (1.18)	5.58 (1.02)	*p* > 0.05	***p*** **< 0.001 F(1, 23.773) = 23.080**
Insecure	Secure	5.77 (0.98)	5.33 (1.28)	***p*** **< 0.05 F(1, 6.214) = 5.435**	5.38 (1.08)	5.38 (0.82)	*p* > 0.05	***p*** **< 0.05 F(1, 6.314) = 5.554**
Incapable	Capable	5.93 (0.82)	5.64 (0.94)	*p* > 0.05	5.24 (1.11)	5.13 (1.15)	*p* > 0.05	***p*** **< 0.001 F(1, 33.544) = 33.320**
Rough	Sensitive	5.05 (1.13)	4.64 (1.03)	***p*** **< 0.05 F(1, 5.383) = 4.431**	4.54 (1.24)	4.33 (0.64)	*p* > 0.05	***p*** **< 0.01 F(1, 15.756) = 11.910**
Impersonal	Friendly	5.25 (1.19)	4.82 (1.03)	***p*** **< 0.05 F(1, 5.945) = 4.532**	4.74 (1.42)	4.61 (0.99)	*p* > 0.05	***p*** **< 0.01 F(1, 14.826) = 9.088**
Cagy	Open	5.17 (1.00)	4.80 (0.98)	***p*** **< 0.05 F(1, 4.413) = 4.445**	4.72 (1.42)	4.63 (0.92)	*p* > 0.05	***p*** **< 0.01 F(1, 11.339) = 7.756**

*AUS* Australia, *GER* Germany

**Table 4:** Summary of ratings of the ‘doctor they will become (self)’

Likert scale (SELF)		Mean (SD)						
1	7	AUS yr1	AUS yr 5	Diff (p)	GER yr1	GER yr 5	Diff (p)	AUS vs GER difference (p)
Indifferent	Understanding	6.69 (0.53)	6.50 (0.62)	***p*** **< 0.001 F(1, 13.069) = 13.806**	5.88 (0.81)	6.13 (0.68)	*p* > 0.05	***p*** **< 0.001 F(1,47.277) = 97.081**
Conservative	Progressive	5.98 (0.98)	5.87 (1.07)	***p*** **< 0.05 F(1, 7.711) = 6.199**	5.18 (1.11)	4.88 (0.99)	*p* > 0.05	***p*** **< 0.001 F(1, 56.954) = 51.335**
Superficial	Thorough	6.64 (0.55)	6.46 (0.72)	***p*** **< 0.01 F(1, 11.041) = 9.856**	5.93 (1.00)	5.50 (0.98)	*p* > 0.05	***p*** **< 0.001 F(1, 44.448) = 62.920**
Powerless	Powerful	5.30 (0.93)	5.33 (0.94)	*p* > 0.05	3.89 (1.12)	3.83 (0.82)	*p* > 0.05	***p*** **< 0.001 F(1, 178.935) = 174.077**
Passive	Active	6.27 (0.74)	6.11 (0.77)	*p* > 0.05	5.34 (1.16)	5.13 (1.08)	*p* > 0.05	***p*** **< 0.001 F(1, 73.380) = 76.479**
Disagreeable	Likeable	6.46 (0.73)	6.43 (0.69)	***p*** **< 0.01 F(1, 5.873) = 7.073**	5.90 (0.84)	5.96 (0.86)	*p* > 0.05	***p*** **< 0.001 F(1, 26.318) = 42.837**
Weak	Confident	6.40 (0.65)	6.20 (0.75)	*p* > 0.05	5.03 (1.15)	4.83 (1.24)	*p* > 0.05	***p*** **< 0.001 F(1, 158.867) = 171.090**
Realistic	Idealistic	3.86 (1.60)	3.76 (1.37)	*p* > 0.05	3.71 (1.58)	4.25 (1.57)	*p* > 0.05	*p* > 0.05
Egoistic	Selfless	6.18 (0.89)	5.76 (0.99)	***p*** **< 0.01 F(1, 16.237) = 10.449**	4.82 (1.16)	4.96 (0.91)	*p* > 0.05	***p*** **< 0.001 F(1, 132.810) = 122.252**
Rich	Poor	3.53 (1.07)	3.33 (0.90)	*p* > 0.05	4.66 (1.27)	4.75 (1.03)	*p* > 0.05	***p*** **< 0.001 F(1, 126.750) = 97.018**
Unreliable	Reliable	6.72 (0.55)	6.78 (0.51)	*p* > 0.05	6.09 (0.91)	5.75 (0.85)	*p* > 0.05	***p*** **< 0.001 F(1, 42,592) = 74.407**
Authoritarian	Democratic	5.67 (1.06)	5.48 (1.13)	*p* < 0.05 F(1, 8.549) = 5.448	5.24 (1.42)	5.50 (1.14)	*p* > 0.05	***p*** **< 0.05 F(1, 10.556) = 6.719**
Untrustworthy	Trustworthy	6.79 (0.43)	6.85 (0.42)	*p* > 0.05	6.36 (0.69)	6.29 (0.75)	*p* > 0.05	***p*** **< 0.001 F(1, 18.496) = 54.095**
Insecure	Secure	6.52 (0.66)	6.41 (0.78)	*p* < 0.05 F(1, 6.214) = 5.435	4.81 (1.32)	4.63 (1.01)	*p* > 0.05	***p*** **< 0.001 F(1, 255.059) = 231.497**
Incapable	Capable	6.83 (0.37)	6.72 (0.46)	*p* > 0.05	5.09 (1.26)	4.92 (0.97)	*p* > 0.05	***p*** **< 001 F(1, 265.333) = 304.682**
Rough	Sensitive	6.08 (0.95)	6.17 (0.82)	***p*** **< 0.05 F(1, 5.383) = 4.431**	5.82 (1.08)	5.92 (0.72)	*p* > 0.05	***p*** **< 0.05 F(1, 6.552) = 6.747**
Impersonal	Friendly	6.60 (0.61)	6.54 (0.59)	***p*** **< 0.05 F(1, 5.945) = 4.532**	6.15 (0.87)	6.00 (0.95)	*p* > 0.05	***p*** **< 0.001 F(1, 18.285) = 31.271**
Cagy	Open	6.36 (0.75)	6.17 (0.88)	***p*** **< 0.05 F(1, 4.413) = 4.445**	5.51 (1.38)	5.75 (0.99)	*p* > 0.05	***p*** **< 0.001 F(1, 51.549) = 41.618**

Here, we present the results for each of the four cohorts on each of the three constructs. In each case, the construct seen as the most positive image of the physician is presented.

### What are student’s perceptions of the ideal doctor?

Overall, the ideal physician is perceived as ‘understanding’, ‘thorough’, ‘reliable’, ‘trustworthy’, ‘likeable’, and ‘capable’. Scores are lower for some items, favouring ‘realistic’, ‘rich’, and ‘authoritarian’. In general, perceptions were similar between cohorts with the exception of some small but significant differences (please see Table 2 for more details). Responses did not differ within country cohorts (between years) apart from ‘egoistic’ versus ‘selfless’ in which first year Australian students favoured ‘selflessness’ compared to final year students (*p* < 0.01) and ‘realistic’ versus ‘idealistic’ in which first year German students favoured ‘realistic’ more than final year students (*p* < 0.05).

### What are student’s perceptions of the real doctor?

Perceptions of the real doctor were markedly different between Australian and German, with significant differences on the majority of items. Among Australian students, a significant shift occurred from first to final year on several items (please see Table 3 for details). No significant differences existed between first and final year German students.

### What are student’s perceptions of themselves or the doctor they hope to become?

Differences between Australian and German students existed for all but one item in their self- reflection. The only item not statistically significantly different was ‘realism’ versus ‘idealism’. While the questions were subtly different between cohorts, the patterns were generally in the same direction as observed for the real doctor, so they may reflect actual differences between cohorts. Some key differences also existed between first and final year for Australian students (please see Table 4 for details). No significant differences existed between first and final year German students.

### Comparison of the three perceptions separated for German and Australian students

**Fig. 4:**
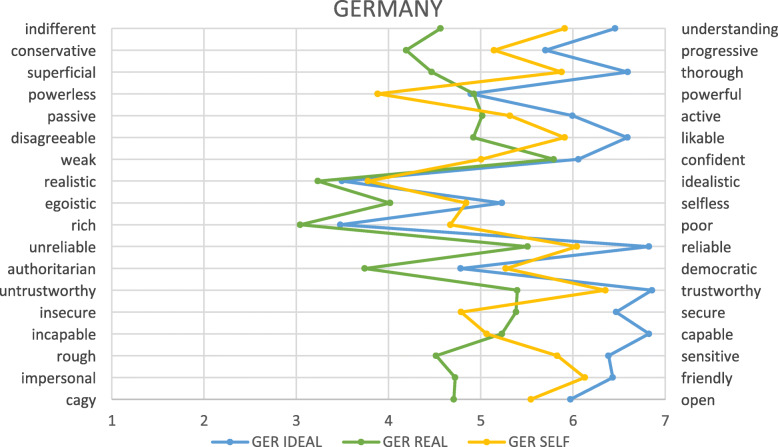
Image of the ideal and real doctor as well as self-image as described by all German students. *AUS = Australia; GER = Germany; numbers relate to year level*

**Fig. 5:**
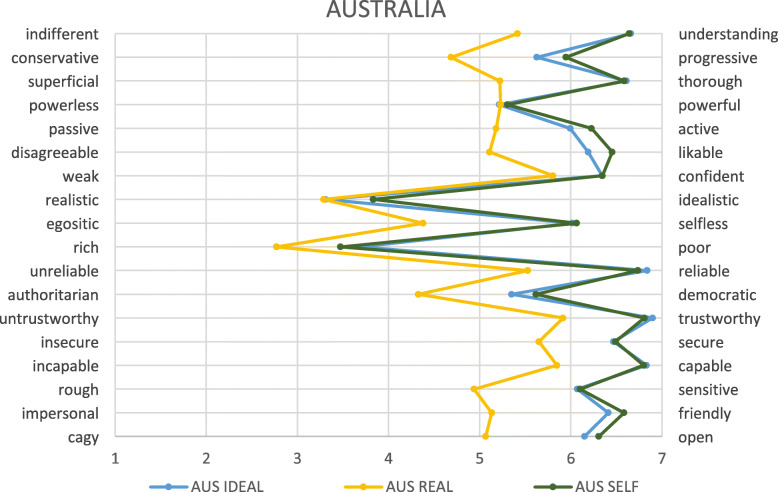
Image of the ideal and real doctor as well as self-image as described by all Australian students. *AUS = Australia; GER = Germany; numbers relate to year level*

In particular, for German students, greater distance was observed between the perceptions of ideal, real, and self, with self-perception laying between the others (see Fig. 4). In the Australian sample, the self-image was closely aligned with the ideal image, and both remained clearly separate from that of the real doctor (see Fig. 5).

## Discussion

In general, the data shows that all four cohorts regarded the ideal doctor very highly, with high scores on core characteristics such as ‘understanding’, ‘thorough’, ‘likeable’, ‘reliable’, ‘trustworthy’, ‘capable’ and ‘friendly’. Scores for the real doctor were lower, with these characteristics perceived to a lesser extent. In the German cohort, self-perception lay between these two, perhaps indicating that personal ideals and values have students striving to be better than that which they perceive as the current reality. In the Australian cohort, students generally perceived themselves as more similar to the ideal doctor rather than the real, which is not surprising given the slight differences in the translated version of the survey. It seems though, that in both cohorts, ideals exist which they do not see emulated in the clinicians to whom they are exposed during their training, and in the case of the German students, which they believe that they are not able to embody.

In general, all cohorts had a similar perception of the ideal doctor, reinforcing the notion that this ideal is a widely held truth about the medical profession. Unlike those relating to the real and self, perceptions of the ideal doctor remained stable from first to final year, and were consistent with values previously identified as desirable by students at the same Australian university [20]. Despite the experiences and understandings of reality which influence change in the other constructs, students retain their beliefs about the fundamental ‘goodness’ of doctors.

In contrast, some differences were observed between Australian and German students, and to a lesser extent between first and final year students in Australia in relation to perceptions of the real doctor. This is likely to reflect cultural differences in the modelling of medical practice in both society as a whole, and in medical education approaches between the two countries. Increasing exposure to hidden curricula factors such as the professional behaviour observed in clinical role-models, organisational pressure such as work hours and pressure to meet key performance indicators may influence students’ perceptions of the real doctor [21].

Differences exist in the medical school cultures between the two countries, including a predominant problem-based and self-directed learning approach in Australia, compared to a more traditional, didactic approach in Germany. In clinical rotations, Australian students are supervised and supported to a variable degree depending on their site and team allocation. While some variation also exists in the German medical school, the majority of faculty associated with the medical school undertake mandatory training in education as part of their induction, offering a higher degree of standardization. These fundamental differences are likely to impact on students’ experiences, and on their perceptions of doctors based on the teachers to whom they are exposed, and the degree of involvement of those teachers.

The most pronounced cross-cultural differences were seen for ‘indifferent’ versus ‘understanding’, ‘conservative’ versus ‘progressive’, ‘superficial’ versus ‘thorough’, ‘authoritarian’ versus ‘democratic’, ‘untrustworthy’ versus ‘trustworthy’ and ‘incapable’ versus ‘capable’. These differences could reflect cultural differences such as those described Hofstede et al. in their 6-dimension model, which enables direct comparisons between countries [22]. Parallels can be drawn between Hofstede’s cultural dimensions and the identity constructs used here, for example; between a ‘progressive’ nature and the concept of ‘indulgence’; between ‘conservatism’ and ‘uncertainty avoidance’, and between ‘understanding’ (as opposed to indifference) and ‘long-term orientation’. This however, does not explain differences in ‘trustworthiness’, ‘capability’, or ‘authoritarianism’. It is possible that cultural differences in these constructs reflect, not a difference in the realities between the cultures, but a difference in the way they are perceived by the students in each cohort. The subjective nature of the polarity scale adds a layer of complexity to the interpretation of these findings.

Several studies of the hidden curriculum describe students’ experiences of becoming more confident and mature in their clinical work and being able to learn from positive role models who emulate knowledge, respect, and patient-centred care [23–26]. These same studies however, report on students’ sense of desensitisation and realisation that the most patient—centred and caring doctors (peers or mentors) are those who become burnt out and jaded [23], and that the hierarchy in medicine often involves humiliation [25]. Further, as students’ improve their time management and efficiency, they reflect on feeling an increasing sense of impatience in both their professional and personal lives [23, 26] and themselves suffer burnout and stress [27, 28]. Silviera et al. describe the culture of ‘speeding up’, where students feel pressured to work quickly and see many patients, as a barrier to the self-reflection and learning required to establish a professional identity. As a result, students find themselves in a state of dissonance between the professional they wanted to be and that which they actually see themselves becoming, triggering shame and guilt [26]. All of these complex components of the hidden curriculum lead to behaviours and values in students which could vary not only at the country or cultural level but within countries at the health service, university, year, or individual level.

The process of medical identity formation is one of socialisation into the profession [10], which likely occurs most profoundly through patient encounters during clinical rotations in the latter years of medical school [29]. In Australia, the change in perceptions of the real doctor over time could reflect negative hidden curricula experiences during medical school, or simply overly idealistic views at the beginning of medical school. Students’ views of the real doctor became more ‘negative’ by final year, with scores reflecting a shift towards doctors as ‘egotistic’, ‘impersonal’, ‘rough’, ‘disagreeable’, and ‘indifferent’. The absence of difference in the German cohorts might mean that the positive hidden curricula influences were stronger for these students, or that they started out with a more realistic perception.

The most pronounced differences in perceptions between cohorts were observed in relation to the image of ‘self’ – but the difference between countries must be interpreted with caution due to the nuances of the question asked. The absence of difference from first to final year somewhat contradicts previous literature suggesting a decline in empathy and altruism [4, 8], but students’ self-perception is not necessarily an accurate reflection of their skill level or success in embodying each of the descriptors used [30]. It is possible that the differences between Australia and Germany are real, aligning with the cultural descriptors of the two countries, with Germany and Australia aligning in some areas and differing markedly in others. For example; Germany as pragmatic and Australia as traditional; Germany as restrained and Australia as optimistic [22]. The pattern observed in the German cohort is consistent with that previously reported among German students [19], but further work will be required to determine whether this pattern is consistent across Australian students more broadly, and when students respond to an identical question about their own identity.

While many of the constructs in the polarity scale reflect the complex processes of socialisation explored above, others are more amenable to ‘intervention’ through educational approaches. Differences between the cohorts were observed in the perception of ‘insecurity’ versus ‘security’, likely closely linked with ‘weakness’ versus ‘confidence’, and ‘incapability’ versus ‘capability’. While these are just a few of the constructs for which the cohorts differed, these reflect important markers of confidence and self-assurance, with the German students consistently rating themselves lower than the Australian students in these measures. These constructs may be a reflection of the difference between self-directed and traditional learning models, and likely indicate students’ sense of unpreparedness for clinical practice [30]. These factors could be addressed though examination of approaches to medical education faculty development to address the hidden curriculum.

This study is limited by small cohorts of students from just two universities in two different countries. The culture of professional identity and the hidden curriculum could vary greatly across universities and of course across other countries. We feel however, that these findings offer a useful starting place for further exploration of identity-formation among medical students in a range of schools and cultures. It is possible that some of the differences between Australian and German students are associated with differences in demographic characteristics such as previous study [20] as well as differences in the admissions process used in these countries. Investigation of these differences is beyond the scope of this study and will be the subject of further research.

Further, the study is limited by low response rates among final year students in both Australia and Germany, and a smaller pool of invited final year respondents in Germany. This is a product of the different recruitment approaches used, with Australian final year students invited largely by email, and only those completing Psychiatry as their final rotation being invited in person. Similarly, in Germany, the final year students invited were those placed at the University Hospital for at least one of their three final year elective placements. While this did limit the participant pool, the fact that the majority of the cohort spend at least one placement at the hospital means that the participants are likely to be a representative sample of their respective cohorts.

## Conclusion

This preliminary study has highlighted some important patterns in medical students’ perceptions which could influence the development of their professionalism and medical identity. Importantly, students’ confidence and sense of security and capability were identified as areas of difference between the cohorts and represent potential targets for educational interventions to improve students’ overall experiences and attitudes. The erosion of self-perception over time is a concern in the context of graduate’s preparedness for practice and the risk of burnout. Further work is needed to better understand the trajectory of change over time in perceptions, and how these constructs relate to students’ and graduates’ performance in assessment and as clinicians. It may be possible to target modifiable constructs among these polarities, in order to improve educational efforts to shape medical identity. Critically, faculty development should be considered as an important target for enhancing the modelling of positive medical identities among those teaching in medical programs, and preventing the erosion in perceptions of the self and the real doctor over time. Medical schools could consider their own perceptions to characterise their desired graduate attributes on a scale such as this, encouraging transparency and efforts towards alignment of perceptions regarding ideal, real and self.

## Data Availability

The datasets generated and/or analysed during the current study are not publicly available but are available from the corresponding author on reasonable request. Detailed data is presented in the tables and figures.

## Abbreviations

BMedSc: Bachelor of Medical Science
MD: Doctor of Medicine
p: *p*-Value
SD: Standard Deviation
SPSS: Statistical Package for the Social Sciences
χ2: Chi-Square Test
